# Paper2GIS: improving accessibility without limiting analytical potential in Participatory Mapping

**DOI:** 10.1007/s10109-022-00386-6

**Published:** 2022-07-01

**Authors:** Timna Denwood, Jonathan J. Huck, Sarah Lindley

**Affiliations:** Department of Geography, University of Manchester, Manchester, UK

**Keywords:** PPGIS, Participatory Mapping, Digital divides, Sketch mapping, C18

## Abstract

Participatory Mapping encompasses a broad spectrum of methods, each with advantages and limitations that can influence the degree to which the target audience is able to participate and the veracity of the data collected. Whilst being an efficient means to gather spatial data, the accessibility of online methods is limited by digital divides. Conversely, whilst non-digital approaches are more accessible to participants, data collected in this way are typically more challenging to analyse and often necessitate researcher interpretation, limiting their use in decision-making. We therefore present ‘Paper2GIS’, a novel sketch mapping tool that automatically extracts mark-up drawn onto paper maps and stores it in a geospatial database. The approach embodied in our tool simultaneously limits the technical burden placed on the participant and generates data comparable to that of a digital system without the subjectivity of manual digitisation. This improves accessibility, whilst simultaneously facilitating spatial analyses that are usually not possible with paper-based mapping exercises. A case study is presented to address two energy planning questions of the residents in the Outer Hebrides, UK. The results demonstrate that accessibility can be improved without impacting the potential for spatial analysis, widening participation to further democratise decision-making.

## Introduction

Participatory Mapping is used as a blanket term to cover a range of participatory methods of gathering spatial data (Brown and Kyttä 2018). It has become a well-established subfield of geography, intended to enhance engagement from historically marginalised groups (Elwood 2006). The inclusion of different parties (and consequently different views and experiences) is widely accepted as being beneficial to the decision-making process, allowing for a more comprehensive understanding of the way citizens interact with space (Huck et al. 2016; Carver et al. 2009; Anderson et al. 2009; Evans and Waters 2007). Participatory Mapping can be used to collect and combine spatial thoughts and ideas from a range of stakeholders to facilitate engagement in policy, decision-making and community level planning (Czepkiewicz et al. 2018; Anderson et al. 2009). It comprises a number of different methodological approaches, including Public Participatory GIS (PPGIS; e.g. Brown 2012) and sketch mapping (e.g. Boschmann and Cubbon 2014), each of which have their own advantages and drawbacks.

The greatest challenges in the successful application of Participatory Mapping are how to achieve the effective representation of the geographic entities about which views are sought, and how to ensure maximum accessibility for the widest possible range of participants (Huck et al. 2017). Indeed, some methods can result in the exclusion of those people they originally set out to empower and therefore fail to effectively represent opinion (Radil and Anderson 2019). Conversely, whilst the use of more accessible, paper-based methods can enable wider participation, they then create difficulties in conducting onward analyses, for instance due to the unstructured nature of the data that are produced (Curtis et al. 2014). This research therefore introduces and demonstrates a novel method of Participatory Mapping to explore whether accessibility for participants can be maximised *whilst* maintaining the capacity for a range of spatial analyses.

### Digital divides

Early forms of Participatory Mapping centred around the use of computing applications and digital visualisations of geospatial data to open up the field of public participation, however, this was often at the expense of wider social and cultural contexts (Dunn 2007; Elwood 2006; Sieber 2006). Subsequently, following the development of Web 2.0, the field of Participatory Mapping grew rapidly, facilitating discussions, encouraging feedback and supporting the decision-making process (Fagerholm et al. 2021; Green 2010; Dunn 2007). Despite web-based approaches meaning that participants can theoretically provide information rapidly, without confrontation and whenever they may choose, it is not always feasible (or desirable) to apply highly technical solutions to elicit responses to spatial questions (Huck et al. 2014). Although there are a number of advantages of using digital approaches for increasing user participation, such as the removal of temporal and geographical limits for participants; their use can also pose challenges. For instance, using digital technologies can exclude those without the skills, inclination, or access to the necessary devices, or the high-speed internet often required to utilise them (Gottwald et al. 2016). In some countries the diffusion of the Internet has reached up to 95%, but as of April 2020 approximately 40% of the global population is still offline, and as such would be excluded from the decision-making process if public participation were only conducted in this way (Statista 2020; van Deursen and van Dijk 2019).

Inequalities in access to digital technologies exist across a broad range of macro and micro-level domains and have previously been referred to as the ‘digital divide’ (Robinson et al. 2015). There is some dispute however over the singularity of the term and the dichotomous image it presents, implying a distinct boundary between the ‘haves’ and ‘have-nots’, which in reality is considerably more nuanced (van Dijk 2020). Instead, it is now considered more appropriate to refer to ‘digital divides’, accounting for the multifaceted and complex reasons behind such inequalities in participation. The issues surrounding digital divides do not necessarily concern digital technology specifically, but rather are inherently connected to socially constructed barriers to access (van Dijk 2020). Digital divides not only reflect social inequalities but can also amplify them, particularly where consultations on public policy and related decisions rely solely on digital methods (Warf 2019). This can lead to inequalities between those who can and cannot participate, in turn resulting in the views of those who have the skills, or means to participate being over-represented (Riddlesden and Singleton 2014; Dunn 2007). Uneven access to the internet and computer materials can occur due to ethnicity (Abreu 2015), gender (Mariscal et al. 2019), education (Crocker and Mazer 2019), disability (Duplaga and Szulc 2019), location (e.g. remote rural areas compared to inner cities; Ye and Yang 2020), and age (Robinson et al. 2015).

Older people are often over-represented in Participatory Mapping studies, yet are also likely to experience accessibility issues with computing equipment (Brown 2017; Haworth et al. 2016; White and Selwyn 2013). Physical challenges such as visual impairments or reduced fine motor skills, as well as more psychological barriers such as technological self-efficacy can make the use of digital technologies more challenging for some older adults (Gottwald et al. 2016; Vrenko and Petrovič 2015; Carpenter and Buday 2007; Nielsen 2013). Although it is not older adults alone that face these challenges—nor all older adults—increasing accessibility to meet these needs can improve the user experience for the wider public as a whole, for example through non-digital methods (Gottwald et al. 2016; Meng and Malczewski 2010). The inclusion of multiple viewpoints across society is central to the benefits and aims of Participatory Mapping, making it important that an appropriate level of accessibility is maintained for the given situation (Radil and Jiao 2016). The challenge, however, is in preventing this from being at the expense of other benefits found with digital methods of Participatory Mapping (i.e. efficiency), or introducing new issues (i.e. the subjectivity of researchers).

### Representations in Participatory Mapping

It is undoubtedly a great technical challenge to translate something as emotive and subjective as public opinion into a tangible form (Godwin and Stasko 2017). Formally, and adopting the terminology of Couclelis (1996); this challenge is how to create digital objects (the GIS representation of a thing) that are capable of adequately representing real-world entities (the thing itself). This is achieved through a combination of an interface (through which the object is created) and a data model (through which the object is stored); with the former providing the focus for this research. Interfaces for Participatory Mapping may be considered to fall into one of two categories. Notative interfaces (based on a scheme of notation), comprise a formalised and pre-agreed mode of communication (e.g. “draw an X where…”), and ensure a consistent scheme of representation between participants. On the other hand, indicative interfaces (based on a scheme of indication), comprise the informal capture and communication of ‘free-hand’ gestures (e.g. “mark the map to show where…”), resulting in high levels of variation between the modes of representation used by individual contributors (after Ingold 2007). When considering notation, Vygotsky (1978) comments that a child who is learning to write cannot truly be said to be writing until they are also capable of reading, as otherwise they are simply reproducing letters without meaningful communication. In this way, notative interfaces in Participatory Mapping (e.g. points, lines and polygons upon which digital map interfaces typically depend), must be both adequately understood by the participant and well suited to the nature of the entity that they are intended to represent, in order to convey a meaningful understanding of the knowledge and views. Indicative interfaces on the other hand are not reliant upon restrictions, however the high levels of variability between individual contributions precludes all but visual comparison.

Digital approaches such as PPGIS typically use complex technology and a strict notation to collect and compile spatial data from a broad range of stakeholders to represent individual interests and priorities on a digital base map (Anderson et al. 2009). The issue of representation is commonly identified in criticisms of the inaccessibility of PPGIS to non-experts (e.g. Godwin and Stasko 2017; Gottwald et al. 2016; Evans and Waters 2007). It has been argued by one author that using point-based notation in particular is a highly effective means of collecting spatial data with PPGIS, yielding high response rates and reducing levels of bias due to the simplicity of the object (Brown 2012). However, point-based data collection methods alone have been criticised for over simplifying the nuanced human experience, restricting complex information to spatially primitive notation and not effectively representing the fundamental characteristics of the entity (Denwood et al. 2020; Huck et al. 2014; Evans and Waters 2007).

Attempts to address this issue in the literature include the use of alternative interfaces or more complex spatial units for the collection of participatory spatial data. Denwood et al. (2020), for example, use a web-based PPGIS but incorporate complex spatial units to provide participants with contextual information in real-time as they input suggestions on the location of a new wind turbine and transport network. Similarly, Huck et al. (2014) created a web-based tool to enable participants to indicate vague areas regarding the positioning of a wind farm, without having to impose ‘artificial boundaries’ (after Montello et al. 2003) onto uncertain areas (a similar approach was also taken by Evans and Waters 2007). Whilst increasing the level of autonomy on the part of the participant and improving the way in which perceptions and ideas are represented as objects, these approaches still require computer technologies and high-speed internet access so in themselves do little to address the digital divides which exclude some from participating.

One solution to this intransient problem may be provided through paper-based methods. ‘Sketch mapping’, for example, is a more accessible method, used to balance the freedom of indication whilst maintaining spatial context through the provision of a paper base map or satellite image (Boschmann and Cubbon 2014). Sketch maps have been used to collect experiential and locational data across a broad range of research areas, such as cycling safety; where children take part in physical activity, and the delineation of neighbourhood boundaries (Marquart et al. 2020; Wridt 2010; and Curtis et al. 2014, respectively). For these purposes, data are often collected at workshops or in small groups to engage with community members and develop dialogue alongside the maps (Wridt 2010; Weiner and Harris 2003). In taking this approach, the use of sketch maps can aid conversation by acting as a visual supplement to qualitative interviews, providing familiarity and comfort for participants in what can sometimes be an intimidating setting, a key challenge in encouraging participants to share information with the researchers (Marquart et al. 2020; Yabiku et al. 2017; Boschmann and Cubbon 2014).

However, whilst the use of sketch maps can improve accessibility, the lack of control over the way in which the maps are marked-up can create difficulties in the resultant spatial analysis. Without a clear scheme of notation, the translation from entity to object can be unclear (Klonner et al. 2018). For example, in Huck et al’s (2017) study, local residents in rural India were asked to indicate what they considered as ‘valuable’ landscape areas. As the participants were given total freedom to mark-up a paper map of the region, the results included wide variations, from the relatively precise (e.g. arrows, crosses and marked routes) to the extremely vague (e.g. a pair of brackets used to indicate a region on the map). Whilst the variety of approaches is certainly interesting for visual analysis, such a range of representations could not be objectively analysed as would be typical with a PPGIS. Similarly, Broelemann et al. (2016) found difficulty with the variation in representation of hand-drawn features, with the mixed representations resulting in their automated digitisation software misclassifying a number of features. Other studies also found the data from sketch maps challenging to digitise and analyse effectively when no consistent approaches were used to mark-up the map (Prener 2020; Pánek et al. 2020). Further, attempts to manually digitise data collected on paper maps can lead to the introduction of subjective interpretation in the resulting dataset due to both the positionality and level of understanding of the researcher (Brown and Kyttä 2018). Whilst flexibility in representation of the entity with sketch mapping gives participants the opportunity to freely illustrate their views and suggestions on the base map, placing more restrictions with a clear scheme of notation (such as only permitting lines or points to be added to denote certain features or locations), could lead to more comparable and homogenous results (Klonner et al. 2018).

## Research aim

The aim of this research is therefore to demonstrate how a novel method of notative sketch mapping might improve accessibility whilst maintaining the capacity for spatial analysis, using a comparative case study in the Outer Hebrides, UK. The proposed system provides a highly accessible ‘pen and paper’ based method for the participant (ensuring it is widely accessible), whilst utilising automatic digitisation coupled with a strict notation to remove reliance on the researcher to interpret mark-up. This limits the impact of subjective interpretation that can be found in the processing of digitising sketch mapping data, whilst maintaining the efficiency and analytic potential of PPGIS data. In doing so, the benefits of each method are preserved and the challenges minimised, improving the veracity and credibility of the resulting dataset so that it might be more acceptable to policy and decision-makers and therefore ensure that local views are effectively represented in the decision-making process (Boschmann and Cubbon 2014).

## Methods

The system is demonstrated through two examples: the first relates to the visual impact of new wind turbines and the second to the design of a new footpath network, using the isles of Barra and Vatersay, Outer Hebrides, UK, as a case study. This section elaborates further on the specific case study, introduces the paper-based system used to collect and compile the participatory data, how this was implemented remotely, and how it will be assessed through visual comparison to the same questions being asked with a facilitated PPGIS.

### Case study

**Fig. 1:**
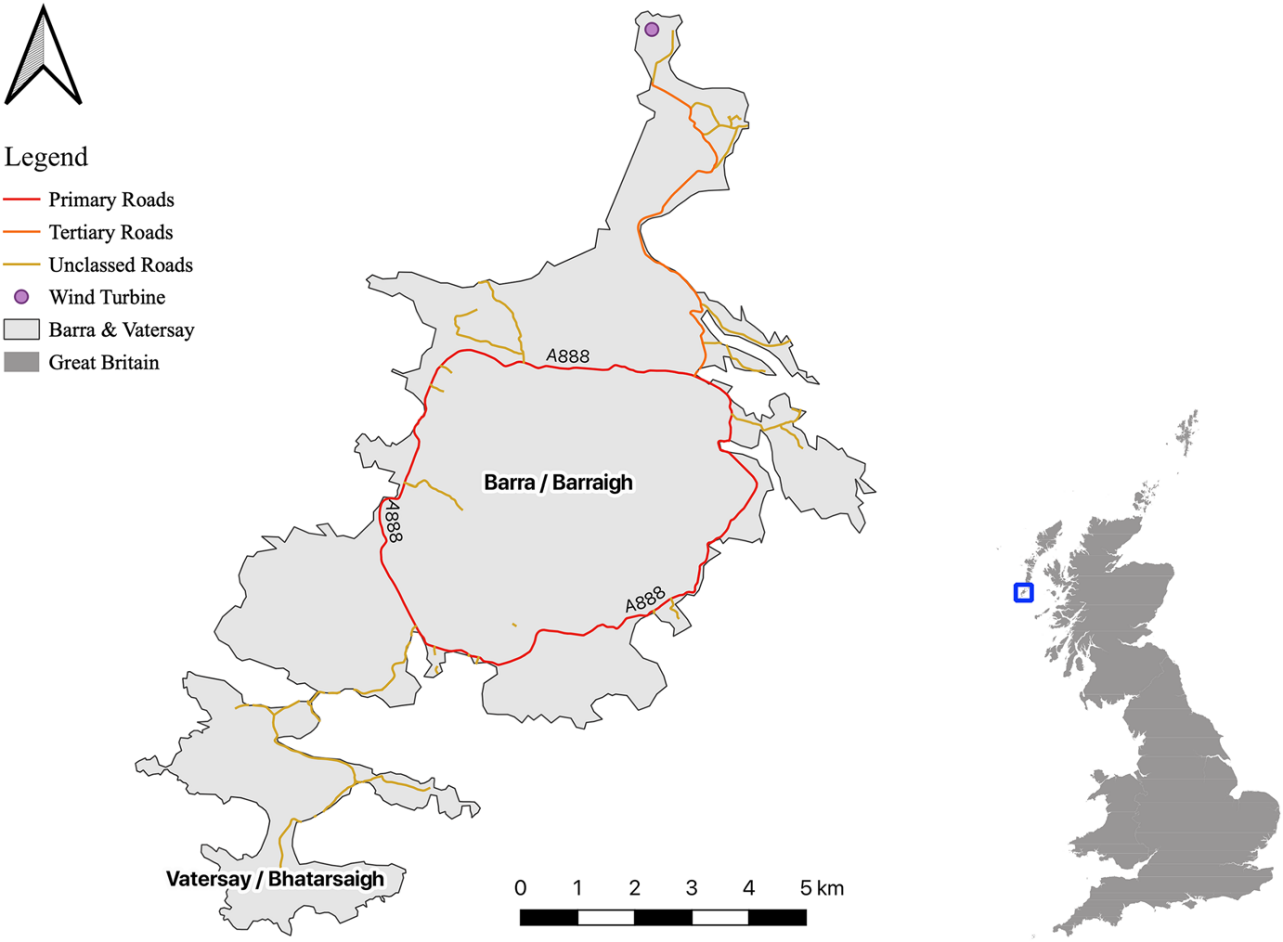
The isles of Barra and Vatersay, Outer Hebrides

The isles of Barra and Vatersay (Fig. 1) cover a total area of approximately 70km2 and host a population of approximately 1300 (CNE Siar 2011). Barra and Vatersay (two separate islands that are joined by a causeway) consist of machair (low-lying grassy plains), hills and lochs, with the majority of the population residing in small hamlets and crofts along the coast, leaving the centre of the isles uninhabited. Residents have recently produced a Local Energy Plan to enable the assessment of existing and future energy needs, therefore opening up opportunities to obtain views on challenges already identified as important by those that live there (Local Energy Scotland 2018). The Local Energy Plan specifically identifies the production of electricity and active transport infrastructure as two key areas of concern. This is largely due to the remote location of the isles as can be seen in Fig. 1, which makes importing fuels of any kind challenging and expensive.

The current energy provision infrastructure on the isles is very restricted, comprising a single mains connection to the grid and requiring oil to be shipped in from mainland Scotland. Just one single track primary road (A888) circumnavigates the island of Barra which, coupled with a very limited number of footpaths and pavements, means that the transport systems on the isles are inadequate. Consequently, this has meant that the isles experience high levels of fuel poverty and forced an over-reliance on motorised transport for travelling short distances (Local Energy Scotland 2018). The addition of further local renewable energy sources coupled with a network of new footpaths (to open up opportunities for movement across the isles) and pavements (along roadsides for the purposes of safety) could alleviate some of the pressure on the current system by addressing the issues raised by residents. Whilst it is clear there is demand for an increased renewable energy supply and development of the current active transport infrastructure, the almost complete absence of any existing industrial landscapes on the isles means that any new developments will have a high impact. This, coupled with an economic reliance on tourism brought about by the prevalence of natural beauty on the isles, means that community input is vital to ensure that the most suitable solutions can be identified.

**Fig. 2:**
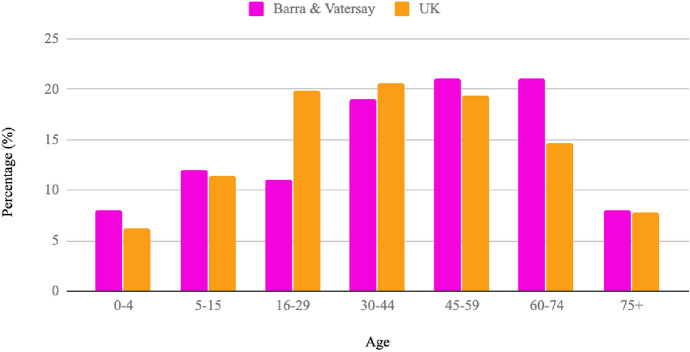
Demographic comparison between % age on the isles of Barra and Vatersay and the UK based on the 2011 Census (data source: Gov.uk 2011; Local Energy Scotland 2018)

In addition to the pre-existing interest in energy challenges, the age demographic of the isles is that of an older population in comparison to the rest of the UK (Fig. 2). Discussion with officials on the isles indicated that the population has since aged further than is represented in the last census, which was carried out almost a decade ago, due to young families moving away from the isles for job opportunities on the mainland. This therefore increases the potential impact that a non-digital Participatory Mapping interface may have on participation in accommodating the needs of older residents.

This location provides a good opportunity to explore the use of non-digital Participatory Mapping interfaces, hosting an older population that could not only benefit from a paper-based method, but is already engaged in the energy challenges facing the isles.

### Data collection

Residents of the isles were asked for their views on two key questions raised by the Local Energy Plan (Local Energy Scotland 2018):

1. From which locations would you not wish to be able to see a wind turbine from on the isles of Barra and Vatersay?
2. Where would you like new footpaths and pavements to be developed on the isles of Barra and Vatersay?

**Fig. 3:**
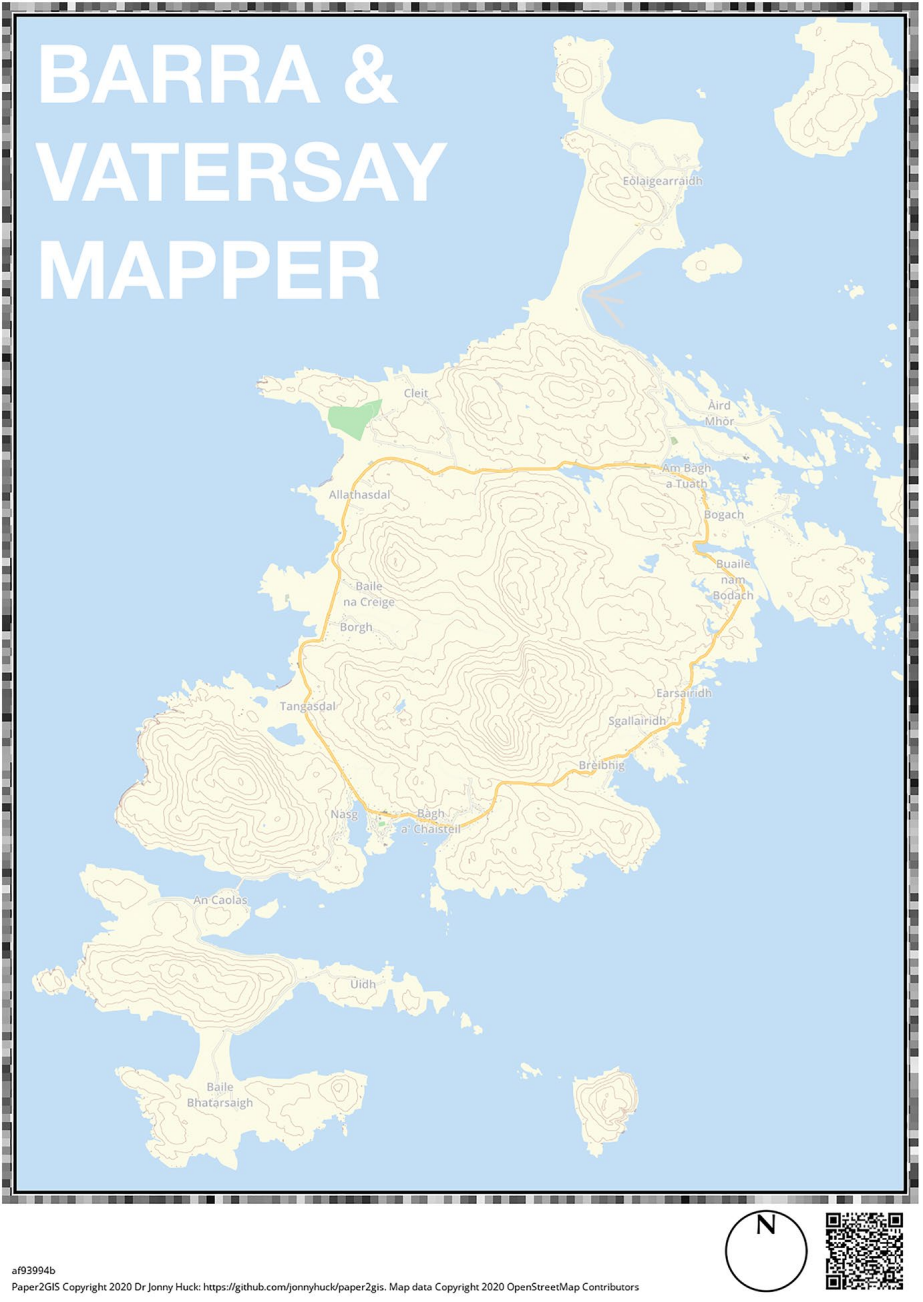
A3 paper map of the isles of Barra and Vatersay used for Paper2GIS data collection

These two spatial energy-planning questions were asked of participants using Paper2GIS, a Participatory Mapping software developed by this research group (https://github.com/jonnyhuck/Paper2GIS). Paper2GIS is used to produce the base map (upon which participants will draw), which includes a QR code containing georeferencing information for the map (bounds and projection information) and a border of random noise Fig. 3; and then automatically extract the markup from the map and export to a GIS. The design of the base map uses the Mapnik renderer (https://mapnik.org), and so is completely customisable using any GIS data source and XML or CSS style sheets. To ensure the methods used are reproducible and transparent as is increasingly encouraged to advance the field (i.e. Brunsdon and Comber 2021; Arribas-Bel et al. 2021) we have followed Denwood et al.’s (2022a) recommendations for an Open Science approach to Participatory Mapping.

**Fig. 4:**
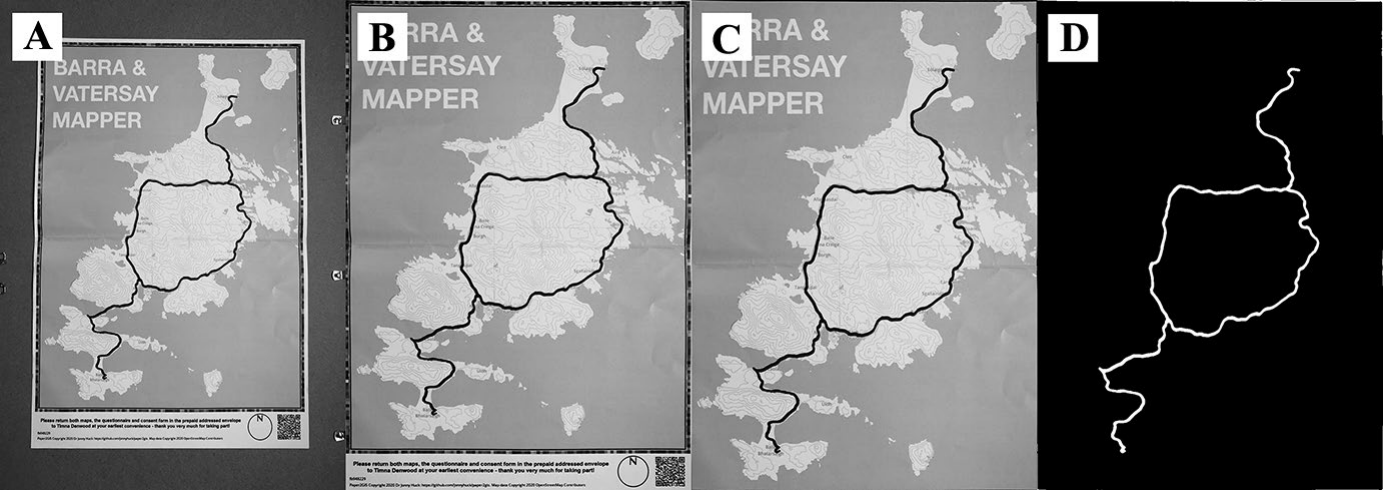
The input and automated extraction using Paper2GIS of a participant-drawn path design that follows the route of the main road on the isles

Markup extraction is achieved using computer vision (CV) technologies in order to allow georeferenced markup to be extracted, cleaned, georeferenced and stored as a GIS dataset using only a photograph of the marked-up map (target image, Fig. 4a) and a ‘clean’ digital version of the map (reference image). Specifically, the paper containing the map is identified in the target image using the SIFT algorithm (Lowe 2004) to detect easily recognisable points in both images, and a FLANN-based matcher is then used to identify matching points of points between the two images, which are evaluated and filtered using Lowe Distance (Lowe 2004) to ensure that spurious matches are rejected. Based upon the relative locations of these matches, image homography calculations (see Malis et al. 2007) are then used to construct a transformation matrix, which may be used to warp the target image in order to correct any perspective distortion caused by differences in the relative planes of the camera and the paper. This results in the extraction and transformation of the paper from the target image (Fig. 4b), which is then cropped to extract the map from the page (Fig. 4c). The extracted map from both the target and reference images are then thresholded (converted to binary image) and ‘differenced’ in order to extract the markup. The resulting markup which is then ‘cleaned’ using image ‘opening’, whereby features in the image are first eroded (making them smaller, which removes noise) and then dilated (which returns the remaining features to their original size). Finally, the markup data are georeferenced using the data extracted from the QR code and exported either as a GeoTiff (GIS raster data format; Fig. 4d) or ShapeFile (GIS vector data format) for analysis.

Although non-digital methods of Participatory Mapping tend to be conducted in a facilitated setting, with adequate instructions they can also be conducted remotely. Therefore, during the COVID-19 restrictions, data collection was conducted via postal delivery. A supply of the required mapping materials along with instructions were sent by post to 525 households to complete and return. The materials consisted of two A3 paper maps, an instruction sheet, a consent form and a feedback questionnaire. The cartographic design used for the basemap included the main roads, water bodies, and main settlements so that participants could locate the areas they wished to mark-up with ease (Fig. 3). Contours were also added so that participants could account for topography when designing the routes for footpaths and pavements. Following both questions, participants were then asked to provide supplementary information using a questionnaire, which allowed contextual data to be collected about the participants, their decisions, and their views on the usability of the non-digital data collection method. Participants were recruited through advertisements on local social media, posters on the isles and in the local newsletter. Participants were neither compensated for their time nor incentivised to participate.

Explicit instructions on notation were given to ensure that the dataset could be analysed without the need for subjective interpretation on the part of the researcher (Klonner et al. 2018). Participants were asked to shade any areas on the map from which they would not wish to be able to see a wind turbine. A similar notative approach has previously been used by Curtis et al. (2014), who asked participants to draw crosses on the map as a notation to represent areas of perceived fear. However, Curtis et al. (2014) identified post-hoc that the varied sizes of resulting crosses still raised questions of representation and interpretation: for example, was the participant referring to the pinpoint location at the centre of the cross, or the whole area that it covered? In order to avoid this issue of uncertainty, shading was selected to allow the participants to clearly and simply show the area they were referring to without the requirement for any further interpretation. This standardised notation enables the shaded areas to be automatically digitised as polygons from each paper map using Paper2GIS and then analysed using viewsheds from the shaded locations. Additionally, by asking participants from where they would rather not be able to see a turbine, participants are being asked something they can reasonably be expected to answer as residents of the isles (Denwood et al. 2020). This contrasts with more traditional approaches to PPGIS surveys, which have asked participants to select already designated areas they deem appropriate for development (e.g. Mekonnen and Gorsevski 2015), which is a more complex and ambiguous question to which a member of the general public could not reasonably be expected to answer.

Participants were then instructed to use a second, identical paper map to draw their desired network of paths on the isles. Drawing lines in digital map interfaces has previously proved difficult in participatory research with non-expert GIS users. Gottwald et al. (2016), for example, removed a line-based tool from their research altogether as it created too many challenges in both representation and accessibility when participants struggled with the high level of technological skill required. Whilst Denwood et al. (2020) sought to address the issue of representation in lines and improve usability through the addition of an underlying routing algorithm, the use of paper-based systems are still more accessible. Marquart et al. (2020) and Yeboah and Alvanides (2015) found that the use of paper-based methods gave participants more confidence in their route-based contributions, without the need for technical knowledge or specific skills.

### Comparison to PPGIS

In order to demonstrate the potential of Paper2GIS as a more accessible interface whilst maintaining the capacity for spatial analysis, the same two questions have also been asked of 22 residents on the isles using a PPGIS (available at https://gitlab.com/timna/informed-interfaces; Denwood et al. 2020). The PPGIS data were collected through facilitated, face-to-face workshops on the isles with a researcher present to provide one-to-one support and instruction throughout. The PPGIS systems make use of underlying algorithms to support the participant in their decision-making by illustrating the implications of their choice in real-time. This was done by firstly calculating viewsheds to delineate areas from which a wind turbine would be visible; and secondly using least-cost-paths between nodes rather than straight ‘point-to-point’ lines to ensure that realistic footpath routes were drawn. Each could be edited before being saved to the database allowing the participant to edit their input accordingly and maintain full control over the output (Denwood et al. 2020).

As both surveys (the PPGIS and Paper2GIS) were conducted independently of one another with relatively small numbers of participants, the results should not be expected to be identical. Nor should one method be considered the benchmark from which the other is assessed, as each has its own advantages and drawbacks as well as being produced by different (yet potentially overlapping) samples of the population. Nevertheless, a simple visual comparison is useful for understanding that similar information can be collected using both methods.

## Results

Overall feedback on the methods of data collection suggests that participants not only understood how to complete the survey, but also felt that collecting data through a paper-based method can enable citizens to be included who may not otherwise be able to when the only option is a digital alternative. Feedback regarding the Paper2GIS survey (which was conducted remotely without additional assistance) included the following comments from participants:

‘Suitable since it is accessible by all who wish to participate whether they have internet access or not and whether mobile or not’ [Female, 61+]

‘It appears to be a good method as the data would be provided by various contributors with different ideas’ [Male, 61+]

Whereas feedback for the PPGIS which was collected during the face-to-face workshops highlighted the issues found amongst older participants with digital interfaces as identified in the literature (e.g. Gottwald et al. 2016) and the importance of having a researcher on hand to assist:

‘Should have brought my glasses, could do with a bigger screen’ [Male, 61+].

‘Good to have help on hand’ [Female, 61+].

### Participants

Between November 2020 and March 2021, 35 households returned the Paper2GIS survey (c.7% of the targeted households). Of these, 23 maps were returned indicating locations from which participants would not wish a wind turbine to be visible. This includes four participants who did not wish to see a wind turbine anywhere on the isles, and seven would be happy to see a turbine anywhere on the isles (and therefore marked no locations on the map). All 35 participants wished to see new footpaths and returned maps indicating where they would prefer them to be routed. Of the 35 households, 91% (*n* = 32) provided the requested demographic information, indicating that 56% (*n* = 18) of those who responded identified as male with the remaining 44% (*n* = 14) identifying as female. Whilst all age groups from 18 years upwards are represented in the respondents, 53% (*n* = 17) were over 61 years of age.

### Unacceptable locations for a new wind turbine

**Fig. 5:**
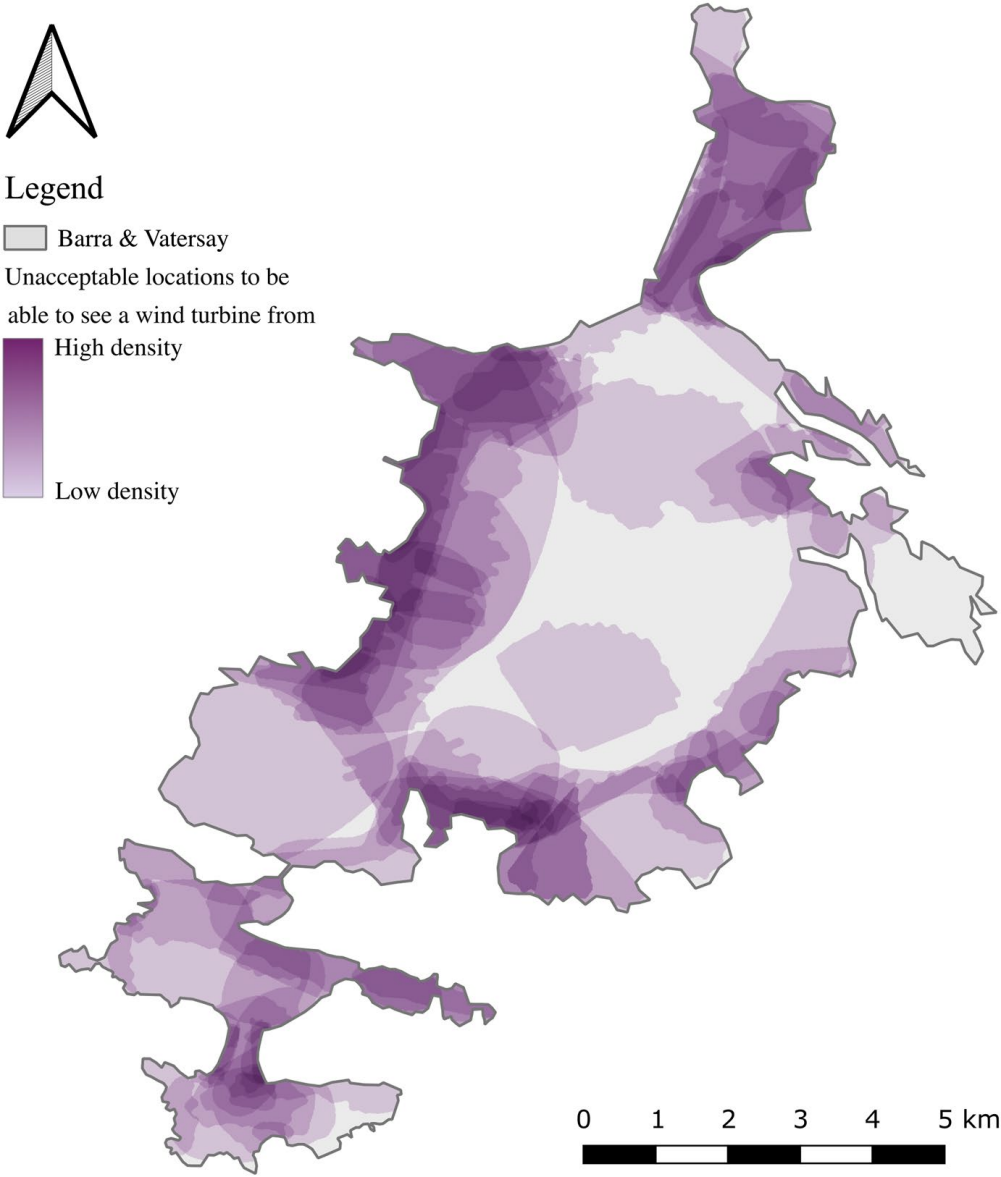
Map indicating locations from where participants would not wish to be able to see a new wind turbine, with darker areas denoting higher levels of agreement

Figure 5 shows the raw dataset (extracted using Paper2GIS) of all areas shaded by residents to delineate areas deemed undesirable from which to be able to see a wind turbine. The darker areas (where multiple shaded areas are overlaid) indicate the most undesirable locations.

**Fig. 6:**
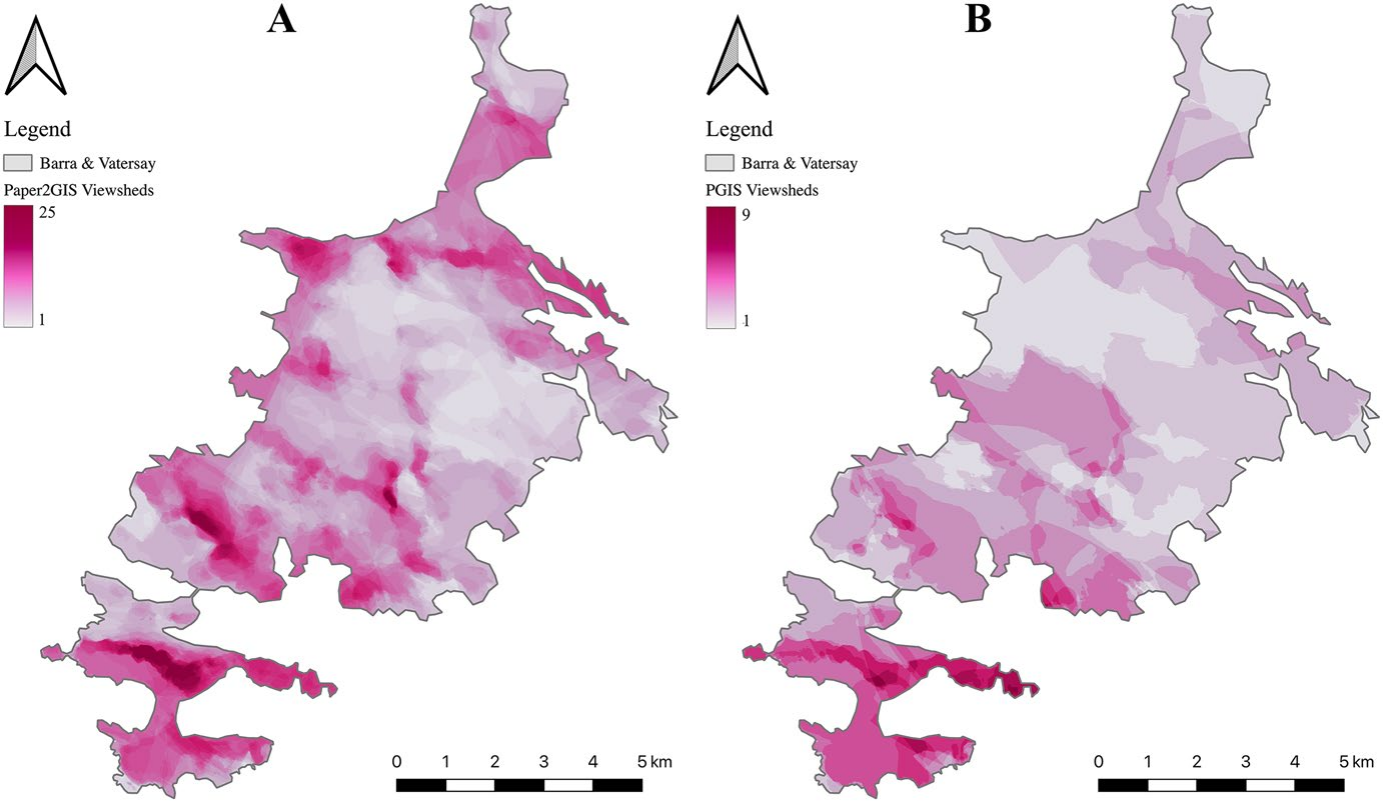
Comparison between viewsheds calculated from the Paper2GIS (**A**) and PPGIS (**B**) datasets produced by independent samples of the population

The shaded areas indicated in Fig. 5 have been converted to viewsheds to enable the visibility of wind turbines in those locations to be analysed by filling each polygon with regular points at 100 m intervals, from each of which a viewshed was calculated. The results of this process are displayed in Fig. 6A. The PPGIS was used to ask the same question of an independent sample, with participants being required to click on a location from which a viewshed would automatically be calculated in real time. The same parameters were used in both cases: 50 m target height (the height of the turbine), 5 km radius (the extent of the viewshed from the turbine location) and 1.6 m observer height (the height of an average person’s eye level).

Figure 6 presents the composite viewshed produced with the dataset from the Paper2GIS (A) results, and the PPGIS (B) results, in which darker areas indicate where multiple viewsheds are overlaid. In this way, the resulting datasets can be considered as an inverse suitability surface, with the darkest colours showing the areas in which a turbine would impact upon the greatest number of the identified locations from which participants would not wish to see a wind turbine. Whilst the magnitude of viewsheds is greater in the Paper2GIS dataset due to the difference in methodology (polygons filled with points, as opposed to single points to represent identified locations) there are similarities in the broad pattern identified in both datasets. Due to these different magnitudes created by the different methodologies, the pink areas are more noticeable in the Paper2GIS example, however the same parts of the island identified as the most unacceptable in both datasets. Such regions include the hills running from East to West across Vatersay (the Southern isle, Fig. 1), the area surrounding the largest settlement (Castlebay, Fig. 1), particularly picturesque peaks and coastlines around the isles, and the area surrounding the airport (located at the North of the main isle, Fig. 1). Participant feedback from the Paper2GIS questionnaire explaining why they did not want to be able to see a wind turbine from certain areas suggested that the primary motivation was in order to maintain the natural beauty of the isles and avoid residential areas:

‘It would spoil the scenery of the area and be harmful to livestock in the area.’ [Male, 61+]

‘Avoiding main areas that are linked to tourist attractions and the views connected.’ [Female, 51–60]

### Desired locations for a new network of paths

**Fig. 7:**
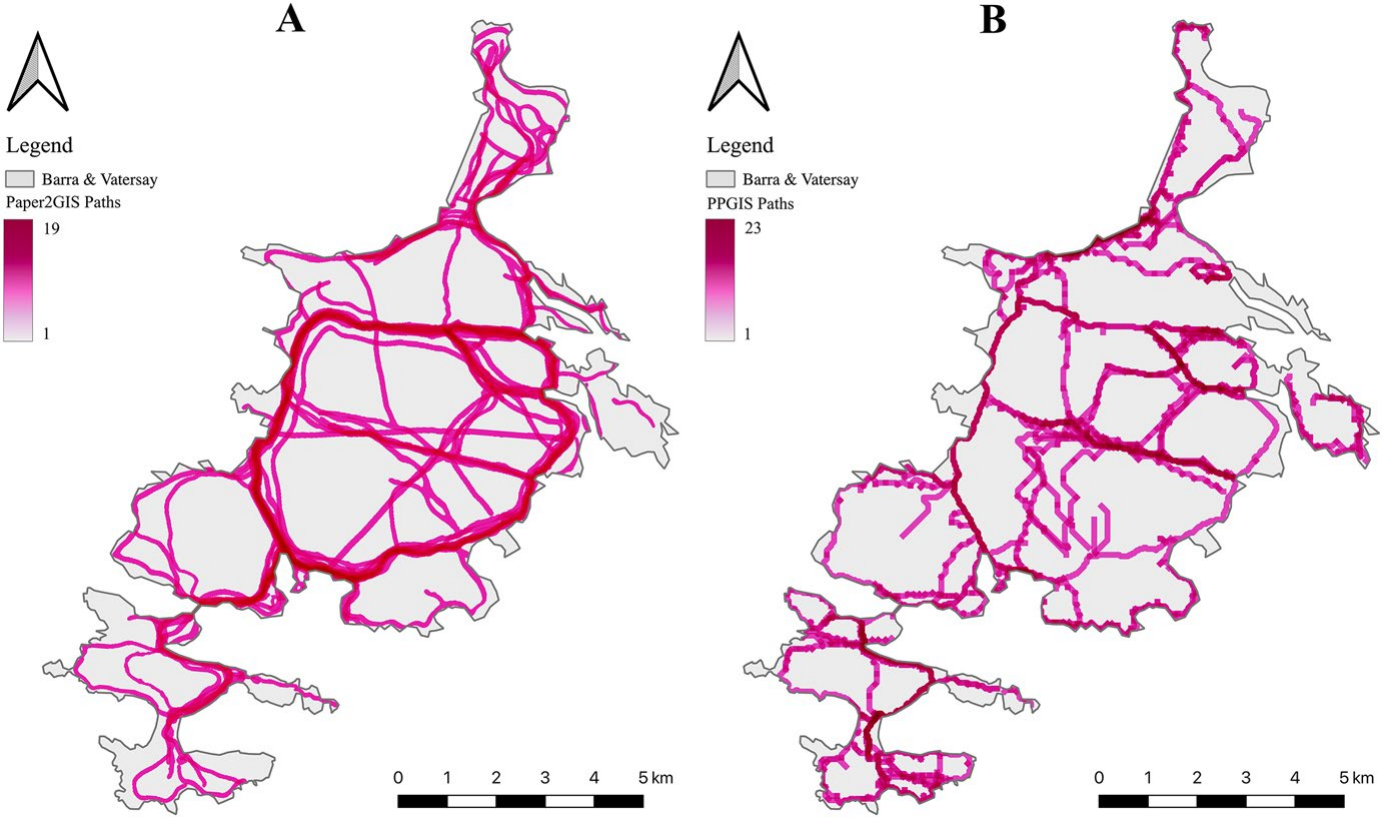
Comparison between the raw network of paths produced from two independent samples, firstly through a Paper2GIS (**A**) and secondly through PPGIS (**B**)

The network of paths proposed by participants using Paper2GIS are presented in Fig. 7A, where darker areas indicate multiple participants proposing the same routes. For example, there is a clear desire from over half of the participants (*n* = 19) for a path alongside the main road which circumnavigates Barra (Fig. 1) to be developed. The comparative dataset is shown in Fig. 7B, which was produced by users clicking a series of points onto the digital map, which were joined together in real time using least cost paths (as opposed to straight lines) in order to give more realistic results.

The results produced through both methods are again broadly similar, giving further confidence that the two datasets are of comparable quality. For example, routes along the coast, as well as through the main valleys running east–west and north–south through the island interior were popular in both datasets. Participant feedback from the Paper2GIS questionnaire suggested a path along the road would be beneficial for safety reasons, whilst the more scenic routes would re-establish and encourage the use of historic walking routes or provide easier access to particular beauty spots:

‘Largely to increase/improve safety and accessibility to existing walks, to increase connectivity of existing path networks, and promote walking for health benefits.’ [Female, 18–30]

‘A footpath would be safer alongside the road from Castlebay to Vatersay Causeway as it is very narrow in places.’ [Male, 61+]

## Discussion

With the growing popularity of Participatory Mapping as a means to improve democracy in the decision-making process, ensuring that methods are accessible to as many diverse members of society as possible is key. The location of the research, the nature of the question being asked and the characteristics of the target population should all be considered when selecting the most appropriate method for any given situation (Denwood et al. 2022a, b).

It is often reported that older people are more likely than those in other age groups to contribute to Participatory Mapping surveys, so the benefits of providing more accessible approaches such as Paper2GIS can be realised in many different contexts, not just those focused on older populations (Haworth et al. 2016). Issues of self-efficacy, visual impairments and reduced mobility (found to limit participation in digital methods of Participatory Mapping) are minimised by using a paper-based method, as is evident in much of the supporting literature (Gottwald et al. 2016; Vrenko and Petrovič 2015; Nielsen 2013). As the population of the isles is generally older than the UK population as a whole, it is likely to benefit from increased accessibility provided by non-digital methods of data collection (Gottwald et al. 2016; White and Selwyn 2013). The similarity in both datasets demonstrates that notative sketch mapping provides a viable alternative or complementary approach to PPGIS for the collection of participatory data, increasing accessibility for those who are unable or unwilling to use digital methods. That a comparable dataset was achieved using Paper2GIS without any facilitation or additional assistance from the researchers suggests that the method and accompanying instructions were easy to understand, and that participants were comfortable using the familiar tools of pen and paper.

Participatory data collected using conventional paper map approaches require digitisation prior to analysis, but the methods to do so are often slow and subject to the interpretation of the researcher (Ramirez-Gomez et al. 2015). The advantage of automated digitisation in Paper2GIS is not only one of time efficiency, enabling accessible participatory research to be carried out at scale and speed; but also an improvement in the replicability of the research by removing the need for manual classification and intermediate interpretations. This also minimises the influence of positionality on the part of the researcher, an ongoing challenge in participatory research (Brown and Kyttä 2018). Due to the prescribed notation, data are comparable to that of a digital equivalent and therefore suitable for a range of spatial analyses rather than only suited to visual analysis.

It is important to recognise that neither dataset (Paper2GIS nor PPGIS) should be considered a benchmark against which the other can be measured, as each approach has its own advantages and drawbacks and there were several differences between the two methods of collection. For example, the fact that one was facilitated, and one remote, along with differences in the characteristics of the participants, the base map, and the instructions will all have influenced the resulting datasets (Curtis et al. 2014). Whilst non-digital methods are more accessible, there are a number of limitations associated with their use in comparison with digital approaches. For example, in using paper maps many of the advantages of digital approaches are lost, such as the ability to zoom in on specific features, switch between map and satellite imagery, and edit data after it has been added to the map. The physical size of the paper base maps in particular can influence the resulting dataset by restricting either the areas covered or the level of detail possible in responses (Yabiku et al. 2017). Whilst increasing the physical size of the maps can improve this situation, by enabling larger geographic areas to be covered at a larger scale (as used by Haworth et al. 2016, Yabiku et al. 2017 and Usher et al. 2020), there is still a trade-off between maintaining accessibility through the simplicity of the system and the advantages of more complex digital systems. However, the similarity between the two datasets in this instance demonstrate that the size and scale of the maps were adequate for the questions being asked, and did not result in any reduction in the quality of data. A further potential limitation could occur in the participants not following or understanding the specific instructions, and, for example, adding alternative mark-up to the maps. This could introduce issues with interpretation of more ambiguous notation (i.e. crosses, circles or brackets), as was encountered by Curtis et al. (2014) and Huck et al. (2017). However, despite the data being collected remotely, no instances of this occurred in this research, with every participant following the set instructions correctly.

Whilst there will always be trade-offs when selecting the most appropriate method, the use of a paper-based approach (coupled with automated digitisation and a prescribed notation), enables the collection of data suitable for spatial analysis whilst simultaneously improving accessibility for the participant. This is vital both for enabling accessible, remote participation and including those for whom participation would be prevented or impacted by digital divides. Although the research presented has focussed on improving accessibility to support an older population in a UK context, Paper2GIS has also been used in other technological contexts where a digital counterpart would not be possible or appropriate. One recent example conducted by this research group is the rapid and wide scale collection of participatory data on COVID-19 transmission in informal settlements in three cities in Kenya. These areas exhibit extremely low levels of access to computers, mobile devices and electricity; and low levels of literacy. Paper2GIS was used to help navigate these challenges in order to support participation from those who would otherwise currently be excluded from the decision-making process. The automated digitisation also facilitated a rapid response, without the inevitable delay that would have been caused by the manual digitisation of > 1200 responses. Another example where Paper2GIS has been successfully used to overcome a different accessibility issue is in the already discussed example in rural India (Huck et al. 2017), which once again demonstrated the benefits of the use of such a technique in a region with no Internet, mobile data or computer access. These examples not only demonstrate the successful deployment of Paper2GIS in a range of contexts but also the potential for future research.

This paper has demonstrated how a notative sketch mapping system can utilise technology to reduce the technical burden placed on the participant and therefore increase accessibility whilst still producing data suitable for spatial analysis. It is self-evident and well understood in the literature that pen and paper is more accessible than digital technology, enabling data to be collected in locations that do not have access to or use of high-speed internet or computer technologies. In turn, this widens the potential for participation, leading to more datasets that are more representative of the stakeholders involved and better-informed decision-making.

## Data Availability

The datasets generated during and/or analysed during the current study are available in the FigShare repositories 10.6084/m9.figshare.17143763 and 10.6084/m9.figshare.17143751.
